# Crystal structure of (*E*)-2-[(2-bromopyridin-3-yl)methyl­idene]-6-meth­oxy-3,4-di­hydro­naphthalen-1(2*H*)-one and 3-[(*E*)-(6-meth­oxy-1-oxo-1,2,3,4-tetra­hydro­naphthalen-2-ylidene)meth­yl]pyridin-2(1*H*)-one

**DOI:** 10.1107/S2056989016009300

**Published:** 2016-06-14

**Authors:** Sarah K. Zingales, Morgan E. Moore, Andrew D. Goetz, Clifford W. Padgett

**Affiliations:** a Armstrong State University, 11935 Abercorn St. Savanah GA 31419, USA

**Keywords:** crystal structure, chalcone, 3-[(*E*)-(6-Meth­oxy-3,4-di­hydro­naphth-2-oyl­idene)meth­yl]-1*H*-pyridin-2-one and (*E*)-2-[(2-bromo-3-pyrid­yl)methyl­idene]-6-meth­oxy-3,4-di­hydro­naphthalen-1-one

## Abstract

The title compounds C_17_H_14_BrNO_2_, (I), and C_17_H_15_NO_3_, (II), were obtained from the reaction of 6-meth­oxy-3,4-di­hydro-2*H*-naphthalen-1-one and 2-bromo­nicotinaldehyde in ethanol. Compound (I) was the expected product and compound (II) was the oxidation product from air exposure. In the crystal structure of compound (I), there are no short contacts or hydrogen bonds. The structure does display π–π inter­actions between adjacent benzene rings and adjacent pyridyl rings. Compound (II) contains two independent mol­ecules, *A* and *B*, in the asymmetric unit; both are non-planar, the dihedral angles between the meth­oxy­benzene and 1*H*-pyridin-2-one mean planes being 35.07 (9)° in *A* and 35.28 (9)°in *B*. In each mol­ecule, the 1*H*-pyridin-2-one unit participates in inter­molecular N—H⋯O hydrogen bonding to another mol­ecule of the same type (*A* to *A* or *B* to *B*). The structure also displays π–π inter­actions between the pyridyl and the benzene rings of non-equivalent mol­ecules (*viz*., *A* to *B* and *B* to *A*).

## Abstract 2

The title compounds C_17_H_14_BrNO_2_ (I), and C_17_H_15_NO_3_ (II), were obtained from the reaction of 6-meth­oxy-3,4-di­hydro-2*H*-naphthalen-1-one and 2-bromo­nicotinaldehyde in ethanol. Compound (I) was the expected product and compound (II) was the oxidation product from air exposure.

## Chemical context   

In order to address the need for new therapeutic agents, medicinal chemists have often looked to nature for inspiration. Our research strategy to synthesize novel compounds considered analogs of the natural product chalcone, which contains two aromatic rings and an α-β-unsaturated ketone. Chalcones, bioactive defense mol­ecules found in plants and used in traditional Chinese medicine, have demonstrated anti­cancer, anti­bacterial, anti­fungal, and anti-inflammatory properties (Nowakowska, 2007 ▸; Katsori *et al.*, 2011 ▸). Chalcones that contain meth­oxy groups (Shenvi *et al.*, 2013 ▸; Bandgar *et al.*, 2010 ▸) and/or pyridine groups (Prasad *et al.*, 2008 ▸; Yee *et al.*, 2005 ▸) have demonstrated activity against a variety of cancer cell lines and anti­biotic-resistant bacteria. Thus, we set out to create a library of chalcones that combine those two functional groups. During the synthesis of the title compound (I) by the Claisen–Schmidt condensation of 6-meth­oxy-3,4-di­hydro-2*H*-naphthalen-1-one and 2-bromo­nicotinaldehyde, two different types of crystals were obtained – those of the desired chalcone (I) and those of the oxidized product (II). The title compound (I) is a chalcone analog of one currently being studied for its potential anti­cancer and anti­bacterial activity [unpublished results].
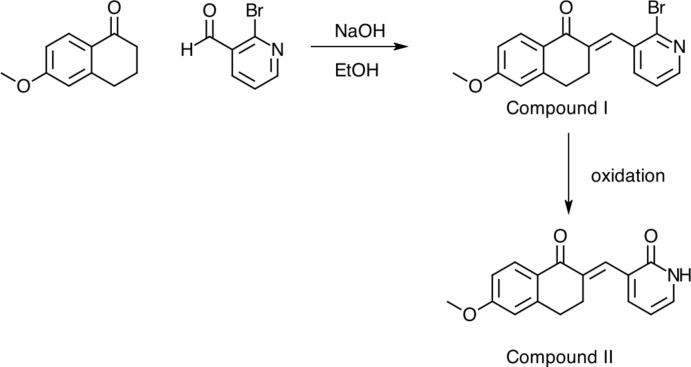


## Structural commentary   

**Figure 1:**
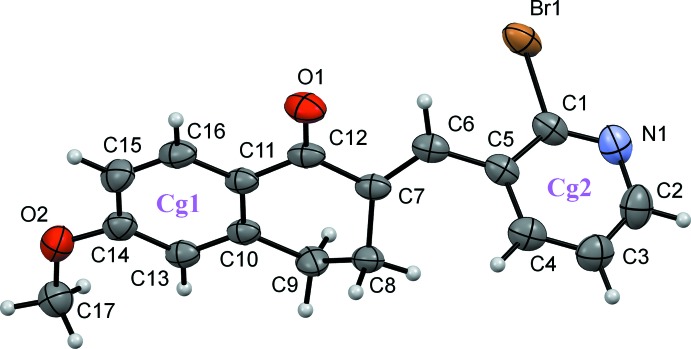
A view of the mol­ecular structure of compound (I), showing the atom and ring labeling. Displacement ellipsoids are drawn at the 50% probability level.

**Figure 2:**
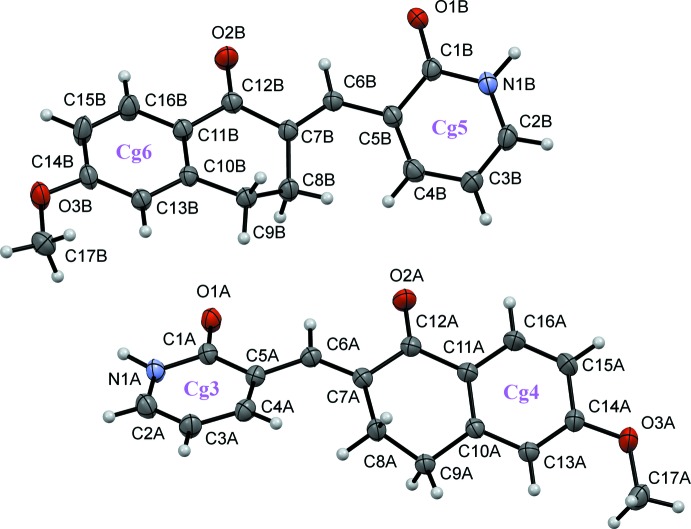
A view of the mol­ecular structure of compound (II), showing the atom and ring labeling. Displacement ellipsoids are drawn at the 50% probability level.

Compound (I) is non-planar (Fig. 1 ▸) with the pyridyl and the benzene ring being rotated by 73.61 (11)°. The C1—Br1 bond distance is 1.916 (4) Å. In compound (II), which presents two independent mol­ecules in the asymmetric unit (*A* and *B*, Fig. 2 ▸), the Br atom is replaced by an oxygen atom, with C—O distances O1*A*—C1*A* = 1.258 (3) and O1*B*—C1*B* = 1.257 (3) Å. The mol­ecules are also non-planar, the benzene–pyridyl angle being 36.18 (10)° in *A* and 35.91 (10)° in *B*.

## Supra­molecular features   

**Figure 3:**
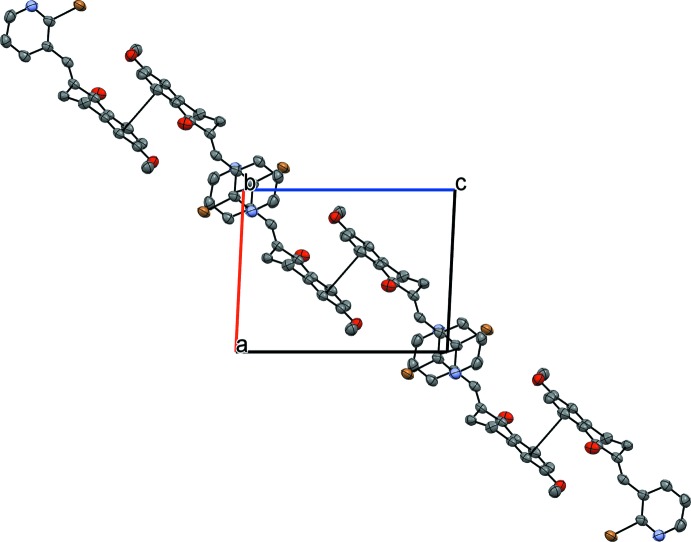
A view of hydrogen-bonded dimers formed in compound (II). Only mol­ecule *A* is shown, for simplicity. Hydrogen bonds (see Table1) are drawn with dashed lines.

**Figure 4:**
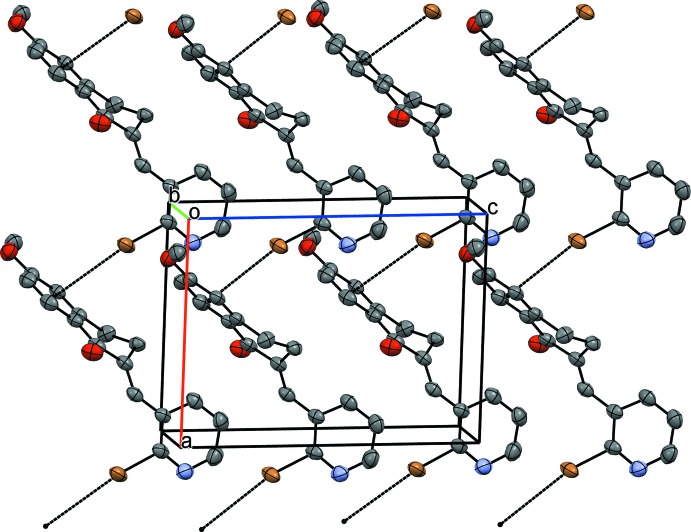
N—H⋯O hydrogen bonding in (II) between 1*H*-pyridin-2-one unit between mol­ecule of the same type

In the crystal structure of (I), mol­ecules are linked by Br⋯π and π–π inter­actions. The Br1⋯*Cg*1^i^ distance is 3.635 (3) Å [symmetry code: (i) −1 + *x*, *y*, *z*; *Cg*1 is the centroid of the benzene ring] and has a ‘face-on’ geometry. There are two π–π inter­actions in the crystal between adjacent benzene rings, *Cg*1⋯*Cg*1^ii^ = 3.944 (4) Å [symmetry code: (ii) 1 − *x*, 1 − *y*, 1 − *z*] and between adjacent pyridyl rings, *Cg*2⋯*Cg*2^iii^ = 3.639 (4) Å [symmetry code: (iii) −*x*, 1 − *y*, −*z*]. The π–π inter­actions form ribbons in the (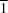
01) plane (Fig. 3 ▸), which are held together by the Br⋯π inter­actions (Fig. 4 ▸).

**Figure 5:**
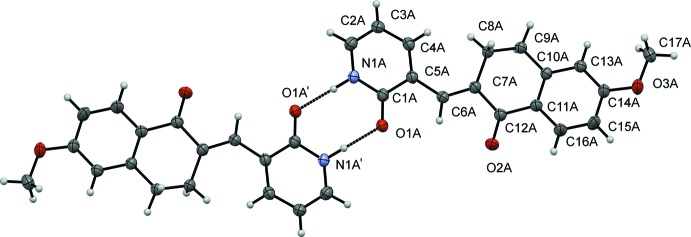
Dimers formed by hydrogen-bonding inter­actions in (II).

**Figure 6:**
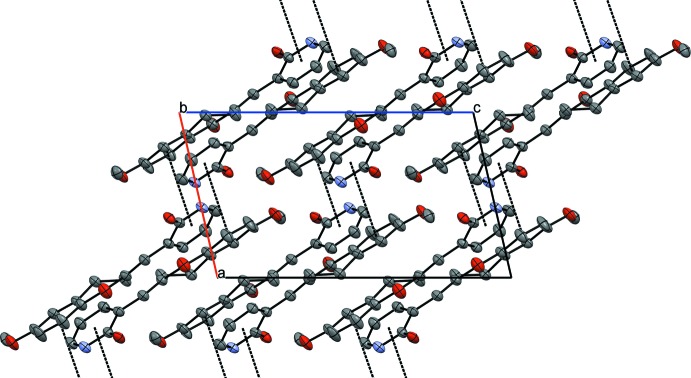
The hydrogen-bonded units in (II) are linked by π–π stacking inter­actions between the phenyl and pyridyl rings in adjacent mol­ecules of different type.

In each one of the independent mol­ecules in (II), the 1*H*-pyridin-2-one unit participates in inter­molecular N—H⋯O hydrogen bonding, with a classical 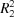 (8) synthon, to another mol­ecule of the same type (*A* to *A* or *B* to *B*), see Fig. 5 ▸ and Table 1 ▸ for details. These hydrogen-bonding inter­actions form dimers that are reminiscent of those frequently observed between carb­oxy­lic acids. The hydrogen-bonded units are linked by π–π stacking inter­actions between the benzene and pyridyl rings in adjacent mol­ecules of different type (*A*–*B* or *B*–*A* inter­actions) (Fig. 6 ▸); *Cg*3⋯*Cg*4^i^ = 3.875 (4) and *Cg*5⋯*Cg*6^ii^ = 3.857 (4) Å [symmetry codes: (i) 3 − *x*, 1 − *y*, −*z*; (ii) 1 − *x*, 1 − *y*, 1 − *z*; *Cg*3 and *Cg*4 are the centroids of the pyridyl and benzene rings of mol­ecule *A*, *Cg*5 and *Cg*6 are the corresponding centroids in mol­ecule *B*].

## Database survey   

A search of the Cambridge Structural Database (Version 5.37 with four updates, Groom *et al.*, 2016 ▸) for structures containing the combined tetra­lone and pyridine backbone returned no hits. The search was broadened by changing the nitro­gen to carbon, which returned 43 hits. The carbon–containing version of (I) has been reported (Dimmock *et al.*, 2002 ▸; Yee *et al.*, 2005 ▸). Many of these similar chalcones also demonstrated biological activities (Dimmock *et al.*, 2002 ▸).

## Synthesis and crystallization   

6-Meth­oxy-3,4-di­hydro-2*H*-naphthalen-1-one (1 mmol) and 2-bromo­nicotinaldehyde (1 mmol) were dissolved in ethanol (5 mL). An NaOH solution (5 M, 1 mL) was added and the reaction was stirred until a precipitate formed. The reaction mixture was cooled in an ice bath for 20 minutes. The solids were filtered off and recrystallized from MeOH/H_2_O. Slow evaporation of a methano­lic solution gave dark purple/brown crystals, which proved to be 3-[(*E*)-(6-meth­oxy-1-oxo-1,2,3,4-tetra­hydro­naphthalen-2-idene)meth­yl]pyridin-2(1*H*)-one, (II), and lighter purple crystals which proved to be (*E*)-2-[(2-bromopyridin-3-yl)methyl­idene]-6-meth­oxy-3,4-di­hydro­naph­thalen-1(2*H*)-one, (I).

## Refinement   

Crystal data, data collection and structure refinement details are summarized in Table 2 ▸. All H atoms were positioned geometrically and refined as riding with C—H = 0.95 or 0.98 Å and *U*_iso_(H) = 1.2*U*_eq_(C).

## Supplementary Material

Crystal structure: contains datablock(s) General, I, II. DOI: 10.1107/S2056989016009300/bg2587sup1.cif

Structure factors: contains datablock(s) I. DOI: 10.1107/S2056989016009300/bg2587Isup2.hkl

Structure factors: contains datablock(s) II. DOI: 10.1107/S2056989016009300/bg2587IIsup3.hkl

CCDC references: 1484124, 1484123

Additional supporting information:  crystallographic information; 3D view; checkCIF report

## Figures and Tables

**Table 1:** Hydrogen-bond geometry (Å, °) for (II)

*D*—H⋯*A*	*D*—H	H⋯*A*	*D*⋯*A*	*D*—H⋯*A*
N1*A*—H1*A*⋯O1*A* ^i^	0.96 (3)	1.82 (3)	2.778 (3)	178 (3)
N1*B*—H1*B*⋯O1*B* ^ii^	0.98 (3)	1.80 (3)	2.778 (3)	176 (3)

Symmetry codes: (i) 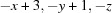; (ii) 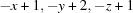.

**Table 2:** Experimental details

	(I)	(II)
Crystal data		
Chemical formula	C_17_H_14_BrNO_2_	C_17_H_15_NO_3_
*M*_r_	344.21	281.31
Crystal system, space group	Monoclinic, *P*2_1_/*c*	Triclinic, *P* 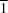
Temperature (K)	173	173
*a*, *b*, *c* (Å)	8.885 (8), 14.253 (13), 11.583 (11)	8.079 (8), 12.296 (12), 14.009 (13)
α, β, γ (°)	90, 92.760 (9), 90	88.85 (3), 76.969 (16), 89.43 (3)
*V* (Å^3^)	1465 (3)	1356 (3)
*Z*	4	4
Radiation type	Mo *K*α	Mo *K*α
μ (mm^−1^)	2.82	0.10
Crystal size (mm)	0.45 × 0.30 × 0.10	0.50 × 0.20 × 0.20

Data collection		
Diffractometer	Rigaku XtaLAB mini	Rigaku XtaLAB mini
Absorption correction	Multi-scan (*REQAB*; Rigaku, 1998 ▸)	Multi-scan (*REQAB*; Rigaku, 1998 ▸)
*T*_min_, *T*_max_	0.587, 0.754	0.808, 0.981
No. of measured, independent and observed [*F* ^2^ > 2.0σ(*F* ^2^)] reflections	15395, 3363, 2583	14459, 6203, 3987
*R*_int_	0.056	0.049
(sin θ/λ)_max_ (Å^−1^)	0.650	0.649

Refinement		
*R*[*F* ^2^ > 2σ(*F* ^2^)], *wR*(*F* ^2^), *S*	0.041, 0.088, 0.99	0.058, 0.157, 1.03
No. of reflections	3363	6203
No. of parameters	190	387
H-atom treatment	H-atom parameters constrained	H atoms treated by a mixture of independent and constrained refinement
Δρ_max_, Δρ_min_ (e Å^−3^)	0.26, −0.46	0.21, −0.23

Computer programs: *CrystalClear-SM Expert* (Rigaku, 2011 ▸), *SHELXS97*, *SHELXS86* and *SHELXL97* (Sheldrick, 2008 ▸) and *CrystalStructure* (Rigaku, 2011 ▸).

## supplementary crystallographic information

### (I) (E)-2-[(2-Bromopyridin-3-yl)methylidene]-6-methoxy-3,4-dihydronaphthalen-1(2H)-one Crystal data

**Table:**

C_17_H_14_BrNO_2_	*F*(000) = 696.00
*M**_r_* = 344.21	*D*_x_ = 1.560 Mg m^−^^3^
Monoclinic, *P*2_1_/*c*	Mo *K*α radiation, λ = 0.71075 Å
Hall symbol: -P 2ybc	Cell parameters from 3472 reflections
*a* = 8.885 (8) Å	θ = 2.3–27.5°
*b* = 14.253 (13) Å	µ = 2.82 mm^−^^1^
*c* = 11.583 (11) Å	*T* = 173 K
β = 92.760 (9)°	Prism, dark-purple/brown
*V* = 1465 (3) Å^3^	0.45 × 0.30 × 0.10 mm
*Z* = 4	

### (I) (E)-2-[(2-Bromopyridin-3-yl)methylidene]-6-methoxy-3,4-dihydronaphthalen-1(2H)-one Data collection

**Table:**

Rigaku XtaLAB mini diffractometer	2583 reflections with *F*^2^ > 2.0σ(*F*^2^)
Detector resolution: 6.827 pixels mm^-1^	*R*_int_ = 0.056
ω scans	θ_max_ = 27.5°
Absorption correction: multi-scan (*REQAB*; Rigaku, 1998)	*h* = −11→11
*T*_min_ = 0.587, *T*_max_ = 0.754	*k* = −18→18
15395 measured reflections	*l* = −15→15
3363 independent reflections	

### (I) (E)-2-[(2-Bromopyridin-3-yl)methylidene]-6-methoxy-3,4-dihydronaphthalen-1(2H)-one Refinement

**Table:**

Refinement on *F*^2^	Secondary atom site location: difference Fourier map
*R*[*F*^2^ > 2σ(*F*^2^)] = 0.041	Hydrogen site location: inferred from neighbouring sites
*wR*(*F*^2^) = 0.088	H-atom parameters constrained
*S* = 0.99	*w* = 1/[σ^2^(*F*_o_^2^) + (0.0211*P*)^2^ + 1.2385*P*] where *P* = (*F*_o_^2^ + 2*F*_c_^2^)/3
3363 reflections	(Δ/σ)_max_ = 0.001
190 parameters	Δρ_max_ = 0.26 e Å^−^^3^
0 restraints	Δρ_min_ = −0.46 e Å^−^^3^
Primary atom site location: structure-invariant direct methods	

### (I) (E)-2-[(2-Bromopyridin-3-yl)methylidene]-6-methoxy-3,4-dihydronaphthalen-1(2H)-one Special details

**Table:**

Refinement. Refinement was performed using all reflections. The weighted R-factor (wR) and goodness of fit (S) are based on F^2^. R-factor (gt) are based on F. The threshold expression of F^2^ > 2.0 sigma(F^2^) is used only for calculating R-factor (gt).

### (I) (E)-2-[(2-Bromopyridin-3-yl)methylidene]-6-methoxy-3,4-dihydronaphthalen-1(2H)-one Fractional atomic coordinates and isotropic or equivalent isotropic displacement parameters (Å^2^)

**Table:**

	*x*	*y*	*z*	*U*_iso_*/*U*_eq_	
Br1	−0.13688 (4)	0.66365 (2)	0.18318 (3)	0.05196 (12)	
O1	0.4068 (3)	0.77510 (13)	0.2922 (2)	0.0510 (6)	
O2	0.8298 (3)	0.48452 (15)	0.56282 (19)	0.0521 (6)	
N1	−0.1337 (3)	0.6115 (2)	−0.0441 (3)	0.0519 (7)	
C1	−0.0406 (4)	0.63829 (19)	0.0420 (3)	0.0399 (7)	
C2	−0.0701 (5)	0.5917 (3)	−0.1437 (3)	0.0569 (9)	
C3	0.0819 (4)	0.5971 (3)	−0.1590 (3)	0.0511 (8)	
C4	0.1750 (4)	0.6256 (2)	−0.0669 (3)	0.0441 (8)	
C5	0.1155 (4)	0.64673 (18)	0.0395 (3)	0.0366 (7)	
C6	0.2119 (4)	0.67894 (19)	0.1378 (3)	0.0382 (7)	
C7	0.3472 (3)	0.64406 (18)	0.1728 (3)	0.0355 (7)	
C8	0.4221 (4)	0.55788 (19)	0.1273 (3)	0.0397 (7)	
C9	0.4650 (4)	0.49170 (19)	0.2271 (3)	0.0431 (7)	
C10	0.5546 (3)	0.53914 (19)	0.3242 (3)	0.0349 (7)	
C11	0.5366 (3)	0.63552 (18)	0.3440 (3)	0.0352 (7)	
C12	0.4297 (3)	0.69188 (19)	0.2712 (3)	0.0380 (7)	
C13	0.6523 (3)	0.48629 (19)	0.3960 (3)	0.0370 (7)	
C14	0.7322 (4)	0.5294 (2)	0.4867 (3)	0.0411 (7)	
C15	0.7157 (4)	0.6258 (3)	0.5061 (3)	0.0477 (8)	
C16	0.6194 (4)	0.6772 (2)	0.4356 (3)	0.0447 (8)	
C17	0.8613 (4)	0.3880 (3)	0.5410 (3)	0.0539 (9)	
H2	−0.1340	0.5728	−0.2077	0.0683*	
H3	0.1219	0.5817	−0.2311	0.0613*	
H4	0.2804	0.6309	−0.0758	0.0530*	
H6	0.1750	0.7297	0.1814	0.0459*	
H8A	0.5135	0.5761	0.0871	0.0476*	
H8B	0.3525	0.5256	0.0710	0.0476*	
H9A	0.3720	0.4651	0.2576	0.0517*	
H9B	0.5248	0.4391	0.1975	0.0517*	
H13	0.6639	0.4210	0.3828	0.0445*	
H15	0.7712	0.6553	0.5682	0.0572*	
H16	0.6088	0.7425	0.4493	0.0536*	
H17A	0.9360	0.3650	0.5993	0.0646*	
H17B	0.9011	0.3816	0.4640	0.0646*	
H17C	0.7684	0.3513	0.5450	0.0646*	

### (I) (E)-2-[(2-Bromopyridin-3-yl)methylidene]-6-methoxy-3,4-dihydronaphthalen-1(2H)-one Atomic displacement parameters (Å^2^)

**Table:**

	*U*^11^	*U*^22^	*U*^33^	*U*^12^	*U*^13^	*U*^23^
Br1	0.04010 (19)	0.0613 (3)	0.0558 (3)	0.01067 (15)	0.01591 (15)	0.01474 (16)
O1	0.0488 (14)	0.0315 (12)	0.0735 (16)	0.0008 (9)	0.0093 (12)	−0.0123 (11)
O2	0.0533 (14)	0.0551 (14)	0.0471 (13)	0.0012 (11)	−0.0050 (11)	−0.0050 (11)
N1	0.0415 (16)	0.0614 (18)	0.0525 (17)	−0.0020 (13)	−0.0003 (14)	0.0138 (14)
C1	0.0361 (16)	0.0365 (16)	0.0479 (18)	0.0030 (12)	0.0094 (14)	0.0138 (13)
C2	0.062 (3)	0.063 (3)	0.045 (2)	−0.0061 (18)	−0.0079 (17)	0.0126 (17)
C3	0.061 (3)	0.051 (2)	0.0415 (18)	0.0011 (16)	0.0087 (16)	0.0097 (15)
C4	0.0470 (19)	0.0387 (17)	0.0478 (19)	−0.0008 (14)	0.0126 (15)	0.0097 (14)
C5	0.0355 (16)	0.0307 (15)	0.0443 (17)	0.0021 (11)	0.0084 (13)	0.0097 (12)
C6	0.0387 (17)	0.0308 (15)	0.0462 (17)	−0.0009 (12)	0.0131 (14)	0.0029 (12)
C7	0.0348 (16)	0.0294 (14)	0.0434 (17)	−0.0018 (11)	0.0130 (13)	−0.0027 (12)
C8	0.0348 (16)	0.0411 (17)	0.0436 (17)	0.0037 (12)	0.0052 (13)	−0.0104 (13)
C9	0.0448 (18)	0.0315 (15)	0.0525 (19)	0.0045 (13)	−0.0029 (15)	−0.0117 (14)
C10	0.0304 (15)	0.0348 (15)	0.0406 (16)	−0.0043 (11)	0.0111 (12)	−0.0073 (12)
C11	0.0301 (15)	0.0323 (14)	0.0439 (17)	−0.0047 (11)	0.0103 (13)	−0.0085 (12)
C12	0.0347 (16)	0.0307 (15)	0.0499 (18)	−0.0049 (12)	0.0162 (13)	−0.0085 (13)
C13	0.0341 (16)	0.0346 (15)	0.0432 (16)	−0.0048 (12)	0.0103 (13)	−0.0059 (13)
C14	0.0366 (16)	0.0471 (18)	0.0404 (17)	−0.0030 (13)	0.0098 (13)	−0.0049 (14)
C15	0.0425 (19)	0.0508 (19)	0.0498 (19)	−0.0093 (15)	0.0016 (15)	−0.0161 (15)
C16	0.0418 (18)	0.0391 (17)	0.0543 (19)	−0.0063 (13)	0.0124 (15)	−0.0162 (14)
C17	0.054 (3)	0.058 (3)	0.049 (2)	0.0080 (17)	0.0013 (16)	0.0003 (16)

### (I) (E)-2-[(2-Bromopyridin-3-yl)methylidene]-6-methoxy-3,4-dihydronaphthalen-1(2H)-one Geometric parameters (Å, º)

**Table:**

Br1—C1	1.916 (4)	C11—C16	1.395 (5)
O1—C12	1.230 (4)	C13—C14	1.383 (5)
O2—C14	1.366 (4)	C14—C15	1.400 (5)
O2—C17	1.428 (4)	C15—C16	1.367 (5)
N1—C1	1.322 (4)	C2—H2	0.950
N1—C2	1.339 (5)	C3—H3	0.950
C1—C5	1.394 (5)	C4—H4	0.950
C2—C3	1.373 (6)	C6—H6	0.950
C3—C4	1.379 (5)	C8—H8A	0.990
C4—C5	1.396 (5)	C8—H8B	0.990
C5—C6	1.466 (4)	C9—H9A	0.990
C6—C7	1.345 (4)	C9—H9B	0.990
C7—C8	1.504 (4)	C13—H13	0.950
C7—C12	1.490 (4)	C15—H15	0.950
C8—C9	1.526 (5)	C16—H16	0.950
C9—C10	1.507 (4)	C17—H17A	0.980
C10—C11	1.403 (4)	C17—H17B	0.980
C10—C13	1.394 (4)	C17—H17C	0.980
C11—C12	1.477 (4)		
			
Br1···C6	3.175 (5)	C7···H17C^vii^	3.4744
O1···C6	2.788 (4)	C8···H2^ix^	3.3343
O1···C16	2.824 (4)	C8···H8A^xii^	3.2064
O2···C16	3.597 (5)	C8···H8B^xii^	3.3383
N1···C4	2.776 (5)	C9···H2^ix^	3.0789
C1···C3	2.682 (5)	C9···H4^xii^	3.4093
C2···C5	2.739 (5)	C10···H3^xii^	3.5613
C4···C7	3.115 (5)	C11···H17C^vii^	3.0608
C4···C8	3.215 (5)	C12···H4^ii^	3.3921
C5···C8	3.129 (5)	C12···H9B^iii^	3.5629
C7···C10	2.898 (5)	C12···H17C^vii^	2.8936
C8···C11	2.883 (5)	C13···H3^xii^	2.9961
C9···C12	2.918 (5)	C14···H9A^vii^	3.1465
C10···C15	2.779 (5)	C14···H17A^iv^	3.4966
C11···C14	2.786 (5)	C14···H17B^iv^	3.5174
C13···C16	2.777 (5)	C15···H2^xi^	3.5940
C13···C17	2.816 (5)	C15···H9A^vii^	3.1588
Br1···C11^i^	3.545 (4)	C15···H17A^iv^	3.3838
O1···C3^ii^	3.482 (5)	C15···H17B^iv^	3.4081
O1···C4^ii^	3.039 (5)	C16···H17C^vii^	3.4878
O1···C9^iii^	3.302 (5)	C17···H6^vi^	3.4285
O2···O2^iv^	3.447 (5)	H2···O2^xiii^	2.9448
O2···C17^iv^	3.549 (5)	H2···C8^ix^	3.3343
C3···O1^v^	3.482 (5)	H2···C9^ix^	3.0789
C4···O1^v^	3.039 (5)	H2···C15^xiii^	3.5940
C5···C17^iii^	3.572 (6)	H2···H8B^ix^	2.9228
C9···O1^vi^	3.302 (5)	H2···H9A^ix^	2.2314
C10···C14^vii^	3.576 (5)	H2···H9B^ix^	3.4838
C11···Br1^viii^	3.545 (4)	H2···H15^xiii^	2.9364
C14···C10^vii^	3.576 (5)	H3···Br1^ix^	3.5417
C17···O2^iv^	3.549 (5)	H3···O1^v^	3.2529
C17···C5^vi^	3.572 (6)	H3···C10^xii^	3.5613
Br1···H6	2.9275	H3···C13^xii^	2.9961
O1···H6	2.4600	H3···H6^v^	2.9190
O1···H16	2.5365	H3···H9B^xii^	3.1583
O2···H13	2.6545	H3···H13^xii^	2.6509
O2···H15	2.4904	H3···H17B^xii^	2.7453
N1···H3	3.2414	H4···O1^v^	2.3578
C1···H2	3.1134	H4···C9^xii^	3.4093
C1···H4	3.2223	H4···C12^v^	3.3921
C1···H6	2.7708	H4···H6^v^	3.5320
C2···H4	3.2239	H4···H8A^xii^	3.4784
C4···H2	3.2145	H4···H9B^xii^	2.4924
C4···H6	3.2363	H4···H16^v^	3.4309
C4···H8A	3.4942	H4···H17C^iii^	3.1940
C4···H8B	2.6124	H6···C3^ii^	3.2152
C5···H3	3.2719	H6···C4^ii^	3.5709
C5···H8B	2.7337	H6···C17^iii^	3.4285
C6···H4	2.6663	H6···H3^ii^	2.9190
C6···H8A	3.1347	H6···H4^ii^	3.5320
C6···H8B	2.6513	H6···H13^iii^	3.1833
C7···H4	2.9181	H6···H17A^vii^	3.0803
C7···H9A	2.7383	H6···H17B^iii^	2.8049
C7···H9B	3.3265	H6···H17C^iii^	3.2031
C8···H4	2.8141	H6···H17C^vii^	3.3881
C8···H6	3.3683	H8A···Br1^viii^	3.4801
C9···H13	2.6622	H8A···N1^viii^	3.5846
C10···H8A	2.8022	H8A···C8^xii^	3.2064
C10···H8B	3.3699	H8A···H4^xii^	3.4784
C10···H16	3.2663	H8A···H8A^xii^	2.9651
C11···H8A	3.0904	H8A···H8B^xii^	2.6613
C11···H9A	2.9829	H8A···H9B^xii^	3.3050
C11···H9B	3.2731	H8A···H16^v^	3.1746
C11···H13	3.2840	H8B···N1^ix^	2.7636
C11···H15	3.2626	H8B···C2^ix^	3.1634
C12···H6	2.5034	H8B···C8^xii^	3.3383
C12···H8A	2.8241	H8B···H2^ix^	2.9228
C12···H8B	3.3631	H8B···H8A^xii^	2.6613
C12···H9A	3.2750	H8B···H8B^xii^	3.2432
C12···H16	2.6445	H8B···H9B^xii^	3.3817
C13···H9A	2.9123	H9A···O1^vi^	3.4120
C13···H9B	2.6028	H9A···O2^vii^	2.9006
C13···H15	3.2694	H9A···N1^ix^	3.3590
C13···H17B	2.7501	H9A···C2^ix^	3.0408
C13···H17C	2.7511	H9A···C14^vii^	3.1465
C14···H16	3.2512	H9A···C15^vii^	3.1588
C14···H17A	3.2009	H9A···H2^ix^	2.2314
C14···H17B	2.6071	H9A···H15^vii^	2.9801
C14···H17C	2.6424	H9B···O1^vi^	2.4164
C15···H13	3.2725	H9B···C3^xii^	3.5819
C17···H13	2.5192	H9B···C4^xii^	3.2624
H2···H3	2.3066	H9B···C12^vi^	3.5629
H3···H4	2.3381	H9B···H2^ix^	3.4838
H4···H6	3.4655	H9B···H3^xii^	3.1583
H4···H8A	2.8438	H9B···H4^xii^	2.4924
H4···H8B	2.3341	H9B···H8A^xii^	3.3050
H6···H8B	3.5753	H9B···H8B^xii^	3.3817
H8A···H9A	2.8675	H9B···H16^vi^	3.4571
H8A···H9B	2.3336	H13···O1^vi^	2.9497
H8B···H9A	2.3265	H13···C3^xii^	3.5284
H8B···H9B	2.4075	H13···H3^xii^	2.6509
H9A···H13	2.9774	H13···H6^vi^	3.1833
H9B···H13	2.4383	H15···Br1^xiv^	2.9998
H13···H17A	3.4907	H15···C1^xiv^	3.4050
H13···H17B	2.3340	H15···H2^xi^	2.9364
H13···H17C	2.2821	H15···H9A^vii^	2.9801
H15···H16	2.3091	H15···H17A^iv^	3.3316
Br1···H3^ix^	3.5417	H15···H17B^iv^	3.0003
Br1···H8A^i^	3.4801	H16···N1^xiv^	3.0906
Br1···H15^x^	2.9998	H16···H4^ii^	3.4309
Br1···H17A^vii^	3.0445	H16···H8A^ii^	3.1746
O1···H3^ii^	3.2529	H16···H9B^iii^	3.4571
O1···H4^ii^	2.3578	H17A···Br1^vii^	3.0445
O1···H9A^iii^	3.4120	H17A···O2^iv^	3.5822
O1···H9B^iii^	2.4164	H17A···C4^vi^	3.5660
O1···H13^iii^	2.9497	H17A···C5^vi^	3.5209
O1···H17C^vii^	3.0841	H17A···C6^vii^	3.4317
O2···H2^xi^	2.9448	H17A···C14^iv^	3.4966
O2···H9A^vii^	2.9006	H17A···C15^iv^	3.3838
O2···H17A^iv^	3.5822	H17A···H6^vii^	3.0803
O2···H17B^iv^	3.0868	H17A···H15^iv^	3.3316
N1···H8A^i^	3.5846	H17B···O2^iv^	3.0868
N1···H8B^ix^	2.7636	H17B···C3^xii^	3.5563
N1···H9A^ix^	3.3590	H17B···C5^vi^	3.3507
N1···H16^x^	3.0906	H17B···C6^vi^	3.2597
C1···H15^x^	3.4050	H17B···C14^iv^	3.5174
C2···H8B^ix^	3.1634	H17B···C15^iv^	3.4081
C2···H9A^ix^	3.0408	H17B···H3^xii^	2.7453
C3···H6^v^	3.2152	H17B···H6^vi^	2.8049
C3···H9B^xii^	3.5819	H17B···H15^iv^	3.0003
C3···H13^xii^	3.5284	H17C···O1^vii^	3.0841
C3···H17B^xii^	3.5563	H17C···C4^vi^	3.2643
C4···H6^v^	3.5709	H17C···C5^vi^	3.2590
C4···H9B^xii^	3.2624	H17C···C6^vi^	3.2537
C4···H17A^iii^	3.5660	H17C···C7^vii^	3.4744
C4···H17C^iii^	3.2643	H17C···C11^vii^	3.0608
C5···H17A^iii^	3.5209	H17C···C12^vii^	2.8936
C5···H17B^iii^	3.3507	H17C···C16^vii^	3.4878
C5···H17C^iii^	3.2590	H17C···H4^vi^	3.1940
C6···H17A^vii^	3.4317	H17C···H6^vi^	3.2031
C6···H17B^iii^	3.2597	H17C···H6^vii^	3.3881
C6···H17C^iii^	3.2537		
			
C14—O2—C17	117.4 (3)	C11—C16—C15	121.2 (3)
C1—N1—C2	115.9 (3)	N1—C2—H2	118.055
Br1—C1—N1	114.2 (3)	C3—C2—H2	118.055
Br1—C1—C5	119.2 (3)	C2—C3—H3	120.838
N1—C1—C5	126.6 (3)	C4—C3—H3	120.843
N1—C2—C3	123.9 (4)	C3—C4—H4	119.759
C2—C3—C4	118.3 (4)	C5—C4—H4	119.753
C3—C4—C5	120.5 (3)	C5—C6—H6	116.619
C1—C5—C4	114.8 (3)	C7—C6—H6	116.628
C1—C5—C6	123.7 (3)	C7—C8—H8A	109.712
C4—C5—C6	121.4 (3)	C7—C8—H8B	109.712
C5—C6—C7	126.8 (3)	C9—C8—H8A	109.713
C6—C7—C8	126.9 (3)	C9—C8—H8B	109.719
C6—C7—C12	117.4 (3)	H8A—C8—H8B	108.195
C8—C7—C12	115.6 (3)	C8—C9—H9A	108.968
C7—C8—C9	109.8 (3)	C8—C9—H9B	108.968
C8—C9—C10	113.1 (3)	C10—C9—H9A	108.972
C9—C10—C11	120.1 (3)	C10—C9—H9B	108.971
C9—C10—C13	119.6 (3)	H9A—C9—H9B	107.765
C11—C10—C13	120.3 (3)	C10—C13—H13	120.158
C10—C11—C12	121.0 (3)	C14—C13—H13	120.170
C10—C11—C16	118.7 (3)	C14—C15—H15	120.081
C12—C11—C16	120.3 (3)	C16—C15—H15	120.079
O1—C12—C7	120.8 (3)	C11—C16—H16	119.393
O1—C12—C11	121.3 (3)	C15—C16—H16	119.404
C7—C12—C11	117.8 (3)	O2—C17—H17A	109.466
C10—C13—C14	119.7 (3)	O2—C17—H17B	109.477
O2—C14—C13	124.7 (3)	O2—C17—H17C	109.473
O2—C14—C15	115.1 (3)	H17A—C17—H17B	109.467
C13—C14—C15	120.3 (3)	H17A—C17—H17C	109.469
C14—C15—C16	119.8 (3)	H17B—C17—H17C	109.476
			
C17—O2—C14—C13	5.8 (4)	C12—C7—C8—C9	50.7 (3)
C17—O2—C14—C15	−174.6 (3)	C7—C8—C9—C10	−52.0 (3)
C1—N1—C2—C3	0.6 (5)	C8—C9—C10—C11	27.9 (4)
C2—N1—C1—Br1	−179.0 (3)	C8—C9—C10—C13	−153.9 (3)
C2—N1—C1—C5	−0.8 (5)	C9—C10—C11—C12	0.4 (4)
Br1—C1—C5—C4	179.22 (15)	C9—C10—C11—C16	179.0 (3)
Br1—C1—C5—C6	−3.3 (4)	C9—C10—C13—C14	−178.6 (3)
N1—C1—C5—C4	1.1 (4)	C11—C10—C13—C14	−0.4 (4)
N1—C1—C5—C6	178.6 (3)	C13—C10—C11—C12	−177.9 (3)
N1—C2—C3—C4	−0.7 (5)	C13—C10—C11—C16	0.8 (4)
C2—C3—C4—C5	1.1 (5)	C10—C11—C12—O1	175.7 (3)
C3—C4—C5—C1	−1.2 (4)	C10—C11—C12—C7	−2.8 (4)
C3—C4—C5—C6	−178.7 (3)	C10—C11—C16—C15	−0.6 (5)
C1—C5—C6—C7	139.3 (3)	C12—C11—C16—C15	178.1 (3)
C4—C5—C6—C7	−43.4 (4)	C16—C11—C12—O1	−2.9 (5)
C5—C6—C7—C8	−7.3 (5)	C16—C11—C12—C7	178.5 (3)
C5—C6—C7—C12	176.3 (3)	C10—C13—C14—O2	179.4 (3)
C6—C7—C8—C9	−125.8 (3)	C10—C13—C14—C15	−0.2 (5)
C6—C7—C12—O1	−25.6 (4)	O2—C14—C15—C16	−179.3 (3)
C6—C7—C12—C11	152.9 (3)	C13—C14—C15—C16	0.4 (5)
C8—C7—C12—O1	157.5 (3)	C14—C15—C16—C11	0.0 (5)
C8—C7—C12—C11	−23.9 (4)		

Symmetry codes: (i) *x*−1, *y*, *z*; (ii) *x*, −*y*+3/2, *z*+1/2; (iii) −*x*+1, *y*+1/2, −*z*+1/2; (iv) −*x*+2, −*y*+1, −*z*+1; (v) *x*, −*y*+3/2, *z*−1/2; (vi) −*x*+1, *y*−1/2, −*z*+1/2; (vii) −*x*+1, −*y*+1, −*z*+1; (viii) *x*+1, *y*, *z*; (ix) −*x*, −*y*+1, −*z*; (x) *x*−1, −*y*+3/2, *z*−1/2; (xi) *x*+1, *y*, *z*+1; (xii) −*x*+1, −*y*+1, −*z*; (xiii) *x*−1, *y*, *z*−1; (xiv) *x*+1, −*y*+3/2, *z*+1/2.

### (II) 3-[(E)-(6-Methoxy-1-oxo-1,2,3,4-tetrahydronaphthalen-2-ylidene)methyl]pyridin-2(1H)-one Crystal data

**Table:**

C_17_H_15_NO_3_	*Z* = 4
*M**_r_* = 281.31	*F*(000) = 592.00
Triclinic, *P*1	*D*_x_ = 1.378 Mg m^−^^3^
Hall symbol: -P 1	Mo *K*α radiation, λ = 0.71075 Å
*a* = 8.079 (8) Å	Cell parameters from 3099 reflections
*b* = 12.296 (12) Å	θ = 1.7–27.5°
*c* = 14.009 (13) Å	µ = 0.10 mm^−^^1^
α = 88.85 (3)°	*T* = 173 K
β = 76.969 (16)°	Prism, purple
γ = 89.43 (3)°	0.50 × 0.20 × 0.20 mm
*V* = 1356 (3) Å^3^	

### (II) 3-[(E)-(6-Methoxy-1-oxo-1,2,3,4-tetrahydronaphthalen-2-ylidene)methyl]pyridin-2(1H)-one Data collection

**Table:**

Rigaku XtaLAB mini diffractometer	3987 reflections with *F*^2^ > 2.0σ(*F*^2^)
Detector resolution: 6.827 pixels mm^-1^	*R*_int_ = 0.049
ω scans	θ_max_ = 27.5°
Absorption correction: multi-scan (*REQAB*; Rigaku, 1998)	*h* = −10→10
*T*_min_ = 0.808, *T*_max_ = 0.981	*k* = −15→15
14459 measured reflections	*l* = −18→18
6203 independent reflections	

### (II) 3-[(E)-(6-Methoxy-1-oxo-1,2,3,4-tetrahydronaphthalen-2-ylidene)methyl]pyridin-2(1H)-one Refinement

**Table:**

Refinement on *F*^2^	Secondary atom site location: difference Fourier map
*R*[*F*^2^ > 2σ(*F*^2^)] = 0.058	Hydrogen site location: inferred from neighbouring sites
*wR*(*F*^2^) = 0.157	H atoms treated by a mixture of independent and constrained refinement
*S* = 1.03	*w* = 1/[σ^2^(*F*_o_^2^) + (0.0585*P*)^2^ + 0.2529*P*] where *P* = (*F*_o_^2^ + 2*F*_c_^2^)/3
6203 reflections	(Δ/σ)_max_ < 0.001
387 parameters	Δρ_max_ = 0.21 e Å^−^^3^
0 restraints	Δρ_min_ = −0.23 e Å^−^^3^
Primary atom site location: structure-invariant direct methods	

### (II) 3-[(E)-(6-Methoxy-1-oxo-1,2,3,4-tetrahydronaphthalen-2-ylidene)methyl]pyridin-2(1H)-one Special details

**Table:**

Refinement. Refinement was performed using all reflections. The weighted R-factor (wR) and goodness of fit (S) are based on F^2^. R-factor (gt) are based on F. The threshold expression of F^2^ > 2.0 sigma(F^2^) is used only for calculating R-factor (gt).

### (II) 3-[(E)-(6-Methoxy-1-oxo-1,2,3,4-tetrahydronaphthalen-2-ylidene)methyl]pyridin-2(1H)-one Fractional atomic coordinates and isotropic or equivalent isotropic displacement parameters (Å^2^)

**Table:**

	*x*	*y*	*z*	*U*_iso_*/*U*_eq_	
O1A	1.35958 (19)	0.48754 (12)	0.11653 (11)	0.0326 (4)	
O1B	0.63793 (19)	0.98881 (12)	0.38219 (11)	0.0332 (4)	
O2A	0.9197 (3)	0.46536 (12)	0.39203 (12)	0.0402 (4)	
O2B	1.0769 (2)	0.96666 (12)	0.10487 (11)	0.0387 (4)	
O3A	0.6064 (3)	0.15815 (13)	0.75027 (11)	0.0442 (5)	
O3B	1.3821 (2)	0.65941 (13)	−0.24660 (11)	0.0412 (5)	
N1A	1.4242 (3)	0.36168 (15)	−0.00284 (13)	0.0298 (4)	
N1B	0.5793 (3)	0.86238 (15)	0.50526 (13)	0.0316 (5)	
C1A	1.3324 (3)	0.39499 (17)	0.08683 (15)	0.0280 (5)	
C1B	0.6684 (3)	0.89632 (17)	0.41435 (15)	0.0278 (5)	
C2A	1.4049 (3)	0.26451 (18)	−0.04361 (17)	0.0354 (6)	
C2B	0.6040 (3)	0.76448 (18)	0.54789 (16)	0.0350 (6)	
C3A	1.2856 (3)	0.19377 (18)	0.00387 (16)	0.0347 (6)	
C3B	0.7238 (3)	0.69400 (18)	0.50207 (16)	0.0342 (6)	
C4A	1.1847 (3)	0.22236 (17)	0.09530 (16)	0.0307 (5)	
C4B	0.8213 (3)	0.72330 (18)	0.40892 (16)	0.0321 (5)	
C5A	1.2066 (3)	0.31888 (17)	0.13941 (15)	0.0272 (5)	
C5B	0.7950 (3)	0.81991 (17)	0.36243 (15)	0.0280 (5)	
C6A	1.1081 (3)	0.35595 (17)	0.23403 (15)	0.0274 (5)	
C6B	0.8909 (3)	0.85753 (17)	0.26640 (15)	0.0270 (5)	
C7A	1.0120 (3)	0.29973 (17)	0.30996 (15)	0.0277 (5)	
C7B	0.9845 (3)	0.80123 (17)	0.19149 (15)	0.0266 (5)	
C8A	0.9851 (3)	0.17786 (17)	0.31644 (15)	0.0326 (5)	
C8B	1.0132 (3)	0.67944 (17)	0.18803 (16)	0.0333 (5)	
C9A	0.9762 (3)	0.13030 (17)	0.41899 (15)	0.0287 (5)	
C9B	1.0185 (3)	0.63272 (17)	0.08736 (15)	0.0283 (5)	
C10A	0.8586 (3)	0.19351 (17)	0.49738 (15)	0.0261 (5)	
C10B	1.1337 (3)	0.69533 (16)	0.00597 (15)	0.0252 (5)	
C11A	0.8353 (3)	0.30523 (17)	0.48528 (15)	0.0288 (5)	
C11B	1.1553 (3)	0.80721 (17)	0.01437 (15)	0.0280 (5)	
C12A	0.9227 (3)	0.36508 (17)	0.39546 (15)	0.0281 (5)	
C12B	1.0721 (3)	0.86650 (17)	0.10352 (15)	0.0279 (5)	
C13A	0.7811 (3)	0.14120 (17)	0.58544 (15)	0.0285 (5)	
C13B	1.2104 (3)	0.64271 (17)	−0.08067 (15)	0.0278 (5)	
C14A	0.6831 (3)	0.20038 (18)	0.66097 (16)	0.0334 (5)	
C14B	1.3057 (3)	0.70229 (18)	−0.15880 (16)	0.0316 (5)	
C15A	0.6584 (4)	0.3122 (2)	0.64862 (18)	0.0454 (7)	
C15B	1.3265 (4)	0.8140 (2)	−0.15060 (17)	0.0421 (7)	
C16A	0.7334 (4)	0.36361 (19)	0.56189 (17)	0.0417 (6)	
C16B	1.2532 (3)	0.86551 (19)	−0.06559 (16)	0.0374 (6)	
C17A	0.6263 (4)	0.04374 (19)	0.76739 (17)	0.0396 (6)	
C17B	1.3661 (4)	0.54477 (19)	−0.25898 (17)	0.0399 (6)	
H1A	1.501 (4)	0.414 (3)	−0.041 (2)	0.054 (8)*	
H1B	0.499 (4)	0.914 (3)	0.543 (2)	0.058 (9)*	
H2A	1.4750	0.2463	−0.1053	0.0424*	
H2B	0.5360	0.7457	0.6106	0.0419*	
H3A	1.2704	0.1259	−0.0242	0.0417*	
H3B	0.7417	0.6263	0.5321	0.0410*	
H4A	1.0986	0.1738	0.1278	0.0368*	
H4B	0.9080	0.6752	0.3770	0.0385*	
H6A	1.1124	0.4321	0.2436	0.0329*	
H6B	0.8865	0.9338	0.2550	0.0324*	
H8A1	1.0793	0.1423	0.2697	0.0391*	
H8A2	0.8781	0.1612	0.2966	0.0391*	
H8B1	0.9209	0.6437	0.2368	0.0400*	
H8B2	1.1217	0.6624	0.2068	0.0400*	
H9A1	0.9368	0.0541	0.4216	0.0344*	
H9A2	1.0915	0.1297	0.4322	0.0344*	
H9B1	1.0579	0.5562	0.0866	0.0340*	
H9B2	0.9022	0.6332	0.0756	0.0340*	
H13A	0.7958	0.0651	0.5935	0.0341*	
H13B	1.1973	0.5665	−0.0860	0.0333*	
H15A	0.5898	0.3524	0.7002	0.0545*	
H15B	1.3918	0.8545	−0.2042	0.0505*	
H16A	0.7160	0.4394	0.5538	0.0501*	
H16B	1.2687	0.9415	−0.0606	0.0449*	
H17A	0.7475	0.0252	0.7539	0.0475*	
H17B	0.5710	0.0026	0.7242	0.0475*	
H17C	0.5738	0.0254	0.8359	0.0475*	
H17D	1.2455	0.5259	−0.2472	0.0479*	
H17E	1.4186	0.5047	−0.2122	0.0479*	
H17F	1.4230	0.5254	−0.3259	0.0479*	

### (II) 3-[(E)-(6-Methoxy-1-oxo-1,2,3,4-tetrahydronaphthalen-2-ylidene)methyl]pyridin-2(1H)-one Atomic displacement parameters (Å^2^)

**Table:**

	*U*^11^	*U*^22^	*U*^33^	*U*^12^	*U*^13^	*U*^23^
O1A	0.0333 (9)	0.0271 (8)	0.0320 (8)	−0.0061 (7)	0.0041 (7)	−0.0016 (7)
O1B	0.0326 (9)	0.0290 (9)	0.0324 (9)	0.0070 (7)	0.0039 (7)	0.0002 (7)
O2A	0.0543 (11)	0.0227 (8)	0.0368 (9)	0.0025 (7)	0.0041 (8)	0.0012 (7)
O2B	0.0513 (11)	0.0219 (8)	0.0369 (9)	−0.0033 (7)	0.0032 (8)	−0.0018 (7)
O3A	0.0536 (11)	0.0373 (10)	0.0307 (9)	0.0040 (8)	0.0132 (8)	0.0055 (8)
O3B	0.0462 (11)	0.0365 (10)	0.0317 (9)	−0.0032 (8)	0.0113 (8)	−0.0075 (7)
N1A	0.0258 (10)	0.0283 (10)	0.0302 (10)	−0.0019 (8)	0.0045 (8)	−0.0008 (8)
N1B	0.0299 (11)	0.0310 (10)	0.0289 (10)	0.0008 (8)	0.0041 (8)	−0.0011 (8)
C1A	0.0240 (11)	0.0277 (11)	0.0298 (11)	0.0010 (9)	−0.0006 (9)	0.0012 (9)
C1B	0.0265 (11)	0.0276 (11)	0.0268 (11)	−0.0036 (9)	−0.0009 (9)	0.0001 (9)
C2A	0.0347 (13)	0.0337 (13)	0.0334 (12)	0.0006 (10)	0.0018 (10)	−0.0062 (10)
C2B	0.0348 (13)	0.0345 (13)	0.0300 (12)	−0.0063 (10)	0.0042 (10)	0.0039 (10)
C3A	0.0417 (14)	0.0251 (12)	0.0331 (12)	−0.0036 (10)	0.0007 (10)	−0.0022 (10)
C3B	0.0383 (13)	0.0278 (12)	0.0328 (12)	0.0007 (10)	−0.0004 (10)	0.0027 (10)
C4A	0.0304 (12)	0.0276 (12)	0.0314 (12)	−0.0044 (9)	−0.0015 (9)	0.0035 (9)
C4B	0.0321 (12)	0.0276 (12)	0.0334 (12)	0.0009 (9)	−0.0001 (10)	−0.0067 (10)
C5A	0.0263 (11)	0.0263 (11)	0.0267 (11)	−0.0002 (9)	−0.0014 (9)	0.0031 (9)
C5B	0.0261 (11)	0.0270 (11)	0.0283 (11)	−0.0001 (9)	−0.0005 (9)	−0.0047 (9)
C6A	0.0295 (12)	0.0222 (11)	0.0285 (11)	−0.0012 (9)	−0.0027 (9)	0.0006 (9)
C6B	0.0277 (11)	0.0224 (10)	0.0283 (11)	0.0004 (8)	−0.0008 (9)	−0.0002 (9)
C7A	0.0261 (11)	0.0255 (11)	0.0286 (11)	0.0026 (9)	−0.0003 (9)	0.0003 (9)
C7B	0.0260 (11)	0.0243 (11)	0.0272 (11)	−0.0015 (8)	−0.0009 (9)	−0.0006 (9)
C8A	0.0371 (13)	0.0258 (12)	0.0283 (12)	0.0003 (9)	0.0067 (10)	−0.0010 (9)
C8B	0.0406 (13)	0.0247 (11)	0.0289 (12)	−0.0018 (10)	0.0044 (10)	−0.0016 (9)
C9A	0.0301 (12)	0.0221 (11)	0.0300 (11)	0.0006 (9)	0.0015 (9)	0.0003 (9)
C9B	0.0304 (12)	0.0218 (11)	0.0300 (11)	−0.0024 (9)	−0.0010 (9)	−0.0005 (9)
C10A	0.0235 (11)	0.0248 (11)	0.0276 (11)	0.0009 (8)	−0.0006 (9)	−0.0010 (9)
C10B	0.0214 (10)	0.0242 (11)	0.0274 (11)	−0.0009 (8)	−0.0004 (9)	0.0001 (9)
C11A	0.0311 (12)	0.0233 (11)	0.0285 (11)	0.0039 (9)	0.0000 (9)	0.0009 (9)
C11B	0.0287 (12)	0.0230 (11)	0.0292 (11)	−0.0044 (9)	0.0002 (9)	−0.0028 (9)
C12A	0.0279 (11)	0.0244 (11)	0.0293 (11)	0.0023 (9)	−0.0010 (9)	0.0017 (9)
C12B	0.0281 (11)	0.0236 (11)	0.0300 (11)	−0.0019 (9)	−0.0022 (9)	−0.0024 (9)
C13A	0.0283 (11)	0.0224 (11)	0.0316 (11)	0.0012 (9)	−0.0004 (9)	0.0008 (9)
C13B	0.0274 (11)	0.0245 (11)	0.0290 (11)	−0.0022 (9)	−0.0010 (9)	−0.0027 (9)
C14A	0.0365 (13)	0.0299 (12)	0.0279 (11)	0.0020 (10)	0.0048 (10)	0.0029 (10)
C14B	0.0289 (12)	0.0334 (12)	0.0273 (11)	−0.0015 (9)	0.0051 (9)	−0.0054 (10)
C15A	0.0554 (17)	0.0346 (14)	0.0347 (13)	0.0151 (12)	0.0137 (12)	−0.0008 (11)
C15B	0.0507 (16)	0.0337 (13)	0.0319 (13)	−0.0136 (11)	0.0122 (11)	−0.0006 (10)
C16A	0.0537 (16)	0.0247 (12)	0.0368 (13)	0.0109 (11)	0.0099 (12)	0.0027 (10)
C16B	0.0450 (15)	0.0276 (12)	0.0333 (12)	−0.0084 (10)	0.0054 (11)	−0.0029 (10)
C17A	0.0469 (15)	0.0341 (13)	0.0335 (13)	−0.0079 (11)	−0.0006 (11)	0.0078 (10)
C17B	0.0470 (15)	0.0342 (13)	0.0355 (13)	0.0066 (11)	−0.0023 (11)	−0.0078 (11)

### (II) 3-[(E)-(6-Methoxy-1-oxo-1,2,3,4-tetrahydronaphthalen-2-ylidene)methyl]pyridin-2(1H)-one Geometric parameters (Å, º)

**Table:**

O1A—C1A	1.258 (3)	C11B—C12B	1.480 (3)
O1B—C1B	1.257 (3)	C11B—C16B	1.405 (3)
O2A—C12A	1.233 (3)	C13A—C14A	1.385 (3)
O2B—C12B	1.233 (3)	C13B—C14B	1.388 (3)
O3A—C14A	1.360 (3)	C14A—C15A	1.400 (4)
O3A—C17A	1.436 (4)	C14B—C15B	1.395 (4)
O3B—C14B	1.360 (3)	C15A—C16A	1.374 (4)
O3B—C17B	1.434 (4)	C15B—C16B	1.369 (4)
N1A—C1A	1.375 (3)	N1A—H1A	0.96 (3)
N1A—C2A	1.360 (4)	N1B—H1B	0.98 (3)
N1B—C1B	1.373 (3)	C2A—H2A	0.950
N1B—C2B	1.366 (4)	C2B—H2B	0.950
C1A—C5A	1.451 (3)	C3A—H3A	0.950
C1B—C5B	1.459 (3)	C3B—H3B	0.950
C2A—C3A	1.354 (4)	C4A—H4A	0.950
C2B—C3B	1.351 (4)	C4B—H4B	0.950
C3A—C4A	1.404 (3)	C6A—H6A	0.950
C3B—C4B	1.406 (4)	C6B—H6B	0.950
C4A—C5A	1.380 (4)	C8A—H8A1	0.990
C4B—C5B	1.381 (4)	C8A—H8A2	0.990
C5A—C6A	1.463 (3)	C8B—H8B1	0.990
C5B—C6B	1.462 (3)	C8B—H8B2	0.990
C6A—C7A	1.349 (3)	C9A—H9A1	0.990
C6B—C7B	1.347 (3)	C9A—H9A2	0.990
C7A—C8A	1.514 (4)	C9B—H9B1	0.990
C7A—C12A	1.496 (3)	C9B—H9B2	0.990
C7B—C8B	1.513 (4)	C13A—H13A	0.950
C7B—C12B	1.497 (3)	C13B—H13B	0.950
C8A—C9A	1.527 (4)	C15A—H15A	0.950
C8B—C9B	1.525 (4)	C15B—H15B	0.950
C9A—C10A	1.504 (3)	C16A—H16A	0.950
C9B—C10B	1.503 (3)	C16B—H16B	0.950
C10A—C11A	1.397 (4)	C17A—H17A	0.980
C10A—C13A	1.398 (3)	C17A—H17B	0.980
C10B—C11B	1.399 (4)	C17A—H17C	0.980
C10B—C13B	1.400 (3)	C17B—H17D	0.980
C11A—C12A	1.482 (3)	C17B—H17E	0.980
C11A—C16A	1.402 (4)	C17B—H17F	0.980
			
O1A···C2A	3.542 (4)	C11A···H3B^vii^	3.4817
O1A···C6A	2.812 (4)	C11A···H4B^vii^	3.1490
O1B···C2B	3.542 (4)	C11B···H2A^i^	3.5524
O1B···C6B	2.821 (4)	C11B···H3A^v^	3.5010
O2A···C6A	2.754 (4)	C11B···H4A^v^	3.1694
O2A···C16A	2.792 (4)	C12A···H3B^vii^	3.1055
O2B···C6B	2.750 (4)	C12A···H17D^v^	3.0137
O2B···C16B	2.798 (4)	C12A···H17F^v^	3.4151
O3B···C16B	3.599 (4)	C12B···H3A^v^	3.2054
N1A···C4A	2.710 (4)	C12B···H17A^vii^	3.0581
N1B···C4B	2.721 (4)	C12B···H17C^vii^	3.4482
C1A···C3A	2.819 (4)	C13A···H1B^iii^	3.28 (4)
C1B···C3B	2.829 (4)	C13A···H4B^vii^	3.5271
C2A···C5A	2.790 (4)	C13A···H9A1^xi^	3.2812
C2B···C5B	2.782 (4)	C13A···H9A2^xi^	3.4733
C4A···C7A	3.177 (4)	C13B···H1A^i^	3.25 (3)
C4A···C8A	3.193 (4)	C13B···H4A^v^	3.5120
C4B···C7B	3.167 (4)	C13B···H9B1^v^	3.2984
C4B···C8B	3.187 (5)	C13B···H9B2^v^	3.5161
C5A···C8A	3.201 (4)	C14A···H2A^viii^	3.3823
C5B···C8B	3.198 (4)	C14A···H4B^vii^	3.5794
C7A···C10A	2.926 (4)	C14A···H8B2^vii^	3.2033
C7B···C10B	2.932 (4)	C14B···H2B^ix^	3.3802
C8A···C11A	2.887 (4)	C14B···H4A^v^	3.5312
C8B···C11B	2.886 (4)	C14B···H8A2^v^	3.1403
C9A···C12A	2.938 (5)	C15A···H2A^viii^	3.5133
C9B···C12B	2.932 (5)	C15A···H4B^vii^	3.4446
C10A···C15A	2.786 (4)	C15A···H8B2^vii^	3.0027
C10B···C15B	2.782 (4)	C15A···H17E^viii^	3.3979
C11A···C14A	2.788 (4)	C15A···H17F^viii^	3.2020
C11B···C14B	2.791 (4)	C15B···H2B^ix^	3.5065
C13A···C16A	2.781 (5)	C15B···H4A^v^	3.3787
C13A···C17A	2.823 (4)	C15B···H8A2^v^	2.9149
C13B···C16B	2.783 (5)	C15B···H17B^xii^	3.2749
C13B···C17B	2.819 (4)	C15B···H17C^xii^	3.2706
O1A···O1A^i^	3.540 (4)	C16A···H3B	3.2475
O1A···O3B^i^	3.528 (4)	C16A···H4B^vii^	3.2293
O1A···N1A^i^	2.778 (3)	C16A···H17F^viii^	3.3113
O1A···C9B	3.366 (4)	C16B···H3A^iv^	3.2754
O1A···C17B^i^	3.316 (5)	C16B···H4A^v^	3.1988
O1B···O1B^ii^	3.556 (4)	C16B···H17C^xii^	3.2954
O1B···O3A^iii^	3.530 (4)	C17A···H2A^viii^	3.1643
O1B···N1B^ii^	2.778 (3)	C17A···H8A1^xi^	3.2466
O1B···C9A^iv^	3.395 (4)	C17A···H15B^xiii^	2.9788
O1B···C17A^iii^	3.343 (5)	C17A···H16B^xiii^	3.5415
O2A···C3B	3.431 (4)	C17B···H2B^ix^	3.1756
O2A···C4B	3.262 (5)	C17B···H8B1^v^	3.2548
O2A···C17B^v^	3.282 (5)	C17B···H15A^ix^	2.9481
O2B···O2B^vi^	3.519 (4)	H1A···O1A^i^	1.82 (3)
O2B···C3A^iv^	3.391 (4)	H1A···N1A^i^	2.93 (3)
O2B···C4A^iv^	3.263 (5)	H1A···C1A^i^	2.71 (3)
O2B···C16B^vi^	3.490 (5)	H1A···C10B^i^	3.36 (3)
O2B···C17A^vii^	3.300 (5)	H1A···C13B^i^	3.25 (3)
O3A···O1B^iii^	3.530 (4)	H1A···H1A^i^	2.42 (4)
O3A···C2A^viii^	3.269 (4)	H1A···H8B2^i^	3.5253
O3B···O1A^i^	3.528 (4)	H1A···H9B1^i^	3.5001
O3B···C2B^ix^	3.277 (4)	H1A···H9B2^v^	3.4543
O3B···C7A^v^	3.522 (5)	H1A···H13B	3.2363
O3B···C12A^v^	3.586 (4)	H1A···H13B^i^	3.3392
N1A···O1A^i^	2.778 (3)	H1A···H17E	2.8349
N1A···C13B^i^	3.411 (5)	H1B···O1B^ii^	1.80 (3)
N1A···C14B^i^	3.554 (5)	H1B···N1B^ii^	2.92 (3)
N1B···O1B^ii^	2.778 (3)	H1B···C1B^ii^	2.70 (3)
N1B···C13A^iii^	3.430 (5)	H1B···C10A^iii^	3.36 (3)
N1B···C14A^iii^	3.584 (5)	H1B···C13A^iii^	3.28 (4)
C1A···C14B^i^	3.494 (5)	H1B···H1B^ii^	2.42 (4)
C1B···C14A^iii^	3.473 (5)	H1B···H8A2^iii^	3.4722
C2A···O3A^ix^	3.269 (4)	H1B···H9A1^iii^	3.4644
C2B···O3B^viii^	3.277 (4)	H1B···H9A2^vii^	3.4365
C3A···O2B^x^	3.391 (4)	H1B···H13A^iv^	3.2562
C3B···O2A	3.431 (4)	H1B···H13A^iii^	3.3828
C3B···C12A^vii^	3.537 (5)	H1B···H15B^viii^	3.5086
C4A···O2B^x^	3.263 (5)	H1B···H17B^iv^	2.9558
C4A···C10B^v^	3.345 (5)	H2A···O1A^i^	3.5344
C4A···C11B^v^	3.461 (5)	H2A···O3A^ix^	2.3436
C4B···O2A	3.262 (5)	H2A···C11B^i^	3.5524
C4B···C10A^vii^	3.331 (5)	H2A···C14A^ix^	3.3823
C4B···C11A^vii^	3.440 (5)	H2A···C15A^ix^	3.5133
C7A···O3B^v^	3.522 (5)	H2A···C17A^ix^	3.1643
C9A···O1B^x^	3.395 (4)	H2A···H8B2^i^	3.4372
C9B···O1A	3.366 (4)	H2A···H9B2^v^	3.3194
C10A···C4B^vii^	3.331 (5)	H2A···H15A^ix^	2.9548
C10B···C4A^v^	3.345 (5)	H2A···H17C^ix^	2.9064
C11A···C4B^vii^	3.440 (5)	H2A···H17E	3.5542
C11B···C4A^v^	3.461 (5)	H2B···O1B^ii^	3.5356
C12A···O3B^v^	3.586 (4)	H2B···O3B^viii^	2.3441
C12A···C3B^vii^	3.537 (5)	H2B···C14B^viii^	3.3802
C12A···C17B^v^	3.493 (5)	H2B···C15B^viii^	3.5065
C12B···C17A^vii^	3.543 (5)	H2B···C17B^viii^	3.1756
C13A···N1B^iii^	3.430 (5)	H2B···H8A2^iii^	3.4956
C13B···N1A^i^	3.411 (5)	H2B···H9A2^vii^	3.3172
C14A···N1B^iii^	3.584 (5)	H2B···H15B^viii^	2.9363
C14A···C1B^iii^	3.473 (5)	H2B···H17F^viii^	2.9233
C14B···N1A^i^	3.554 (5)	H3A···O2B^x^	2.8622
C14B···C1A^i^	3.494 (5)	H3A···O2B^v^	3.4629
C16B···O2B^vi^	3.490 (5)	H3A···C7B^v^	3.5482
C17A···O1B^iii^	3.343 (5)	H3A···C11B^v^	3.5010
C17A···O2B^vii^	3.300 (5)	H3A···C12B^v^	3.2054
C17A···C12B^vii^	3.543 (5)	H3A···C16B^x^	3.2754
C17B···O1A^i^	3.316 (5)	H3A···H9B2^v^	3.3943
C17B···O2A^v^	3.282 (5)	H3A···H16B^x^	2.3352
C17B···C12A^v^	3.493 (5)	H3A···H17C^ix^	3.0415
O1A···H1A	2.43 (3)	H3B···O2A	2.9423
O1A···H6A	2.4489	H3B···O2A^vii^	3.3296
O1B···H1B	2.45 (3)	H3B···C7A^vii^	3.4330
O1B···H6B	2.4642	H3B···C11A^vii^	3.4817
O2A···H6A	2.3405	H3B···C12A^vii^	3.1055
O2A···H16A	2.4985	H3B···C16A	3.2475
O2B···H6B	2.3395	H3B···H9A2^vii^	3.3936
O2B···H16B	2.5041	H3B···H16A	2.3163
O3A···H13A	2.6507	H3B···H17F^viii^	3.1267
O3A···H15A	2.4872	H4A···O2B^x^	2.5858
O3B···H13B	2.6483	H4A···C10B^v^	3.3216
O3B···H15B	2.4886	H4A···C11B^v^	3.1694
N1A···H3A	3.2111	H4A···C13B^v^	3.5120
N1B···H3B	3.2243	H4A···C14B^v^	3.5312
C1A···H2A	3.2667	H4A···C15B^v^	3.3787
C1A···H4A	3.2935	H4A···C16B^v^	3.1988
C1A···H6A	2.5404	H4A···H17A^xi^	3.3142
C1B···H2B	3.2618	H4B···O2A	2.5865
C1B···H4B	3.3034	H4B···C10A^vii^	3.3067
C1B···H6B	2.5497	H4B···C11A^vii^	3.1490
C2A···H4A	3.2254	H4B···C13A^vii^	3.5271
C2B···H4B	3.2206	H4B···C14A^vii^	3.5794
C3A···H1A	3.21 (3)	H4B···C15A^vii^	3.4446
C3B···H1B	3.23 (3)	H4B···C16A^vii^	3.2293
C4A···H2A	3.2394	H4B···H17D^v^	3.4945
C4A···H6A	3.3079	H6A···C8B	3.2615
C4A···H8A1	2.5782	H6A···C9B	3.4562
C4A···H8A2	3.3840	H6A···H8B1	3.0246
C4B···H2B	3.2327	H6A···H8B2	2.8674
C4B···H6B	3.3041	H6A···H9B1	2.7650
C4B···H8B1	2.5740	H6A···H17D^v^	2.9212
C4B···H8B2	3.3796	H6B···C8A^iv^	3.2921
C5A···H1A	3.26 (3)	H6B···C9A^iv^	3.5559
C5A···H3A	3.2879	H6B···H8A1^iv^	3.0467
C5A···H8A1	2.8540	H6B···H8A2^iv^	2.8645
C5A···H8A2	3.5979	H6B···H9A1^iv^	2.8936
C5B···H1B	3.29 (3)	H6B···H15B^vi^	3.5873
C5B···H3B	3.2916	H6B···H16B^vi^	3.5601
C5B···H8B1	2.8515	H6B···H17A^vii^	2.9790
C5B···H8B2	3.5983	H8A1···O2B^x^	3.1964
C6A···H4A	2.7250	H8A1···C17A^xi^	3.2466
C6A···H8A1	2.6674	H8A1···H6B^x^	3.0467
C6A···H8A2	3.0367	H8A1···H13A^xi^	3.4374
C6B···H4B	2.7201	H8A1···H17A^xi^	2.4631
C6B···H8B1	2.6704	H8A1···H17B^xi^	3.3408
C6B···H8B2	3.0366	H8A1···H17C^xi^	3.5252
C7A···H4A	2.9547	H8A2···O1B^x^	2.9375
C7A···H9A1	3.3699	H8A2···O3B^v^	3.2070
C7A···H9A2	2.8359	H8A2···C14B^v^	3.1403
C7B···H4B	2.9446	H8A2···C15B^v^	2.9149
C7B···H9B1	3.3674	H8A2···H1B^iii^	3.4722
C7B···H9B2	2.8279	H8A2···H2B^iii^	3.4956
C8A···H4A	2.5955	H8A2···H6B^x^	2.8645
C8A···H6A	3.3662	H8A2···H15B^v^	2.7849
C8B···H4B	2.5908	H8B1···O2A	3.0557
C8B···H6B	3.3663	H8B1···C17B^v^	3.2548
C9A···H13A	2.6607	H8B1···H6A	3.0246
C9B···H13B	2.6681	H8B1···H17D^v^	2.4760
C10A···H8A1	3.3531	H8B1···H17E^v^	3.3942
C10A···H8A2	2.8177	H8B1···H17F^v^	3.4716
C10A···H16A	3.2727	H8B2···O1A	2.9740
C10B···H8B1	3.3536	H8B2···O3A^vii^	3.2863
C10B···H8B2	2.8138	H8B2···C14A^vii^	3.2033
C10B···H16B	3.2734	H8B2···C15A^vii^	3.0027
C11A···H8A2	3.1634	H8B2···H1A^i^	3.5253
C11A···H9A1	3.2766	H8B2···H2A^i^	3.4372
C11A···H9A2	2.9629	H8B2···H6A	2.8674
C11A···H13A	3.2772	H8B2···H15A^vii^	2.9180
C11A···H15A	3.2692	H9A1···O1B^x^	2.7271
C11B···H8B2	3.1615	H9A1···C1B^x^	2.9439
C11B···H9B1	3.2767	H9A1···C5B^x^	3.2986
C11B···H9B2	2.9529	H9A1···C6B^x^	3.3610
C11B···H13B	3.2834	H9A1···C9A^xi^	3.3310
C11B···H15B	3.2680	H9A1···C13A^xi^	3.2812
C12A···H6A	2.4573	H9A1···H1B^iii^	3.4644
C12A···H8A1	3.3660	H9A1···H6B^x^	2.8936
C12A···H8A2	2.9520	H9A1···H9A1^xi^	2.9166
C12A···H9A2	3.2682	H9A1···H9A2^xi^	2.9945
C12A···H16A	2.6312	H9A1···H13A^xi^	2.5694
C12B···H6B	2.4593	H9A1···H17A^xi^	3.2732
C12B···H8B1	3.3639	H9A2···N1B^vii^	2.9853
C12B···H8B2	2.9405	H9A2···C1B^vii^	3.2133
C12B···H9B2	3.2609	H9A2···C2B^vii^	2.8657
C12B···H16B	2.6379	H9A2···C3B^vii^	2.9221
C13A···H9A1	2.6018	H9A2···C4B^vii^	3.1002
C13A···H9A2	2.9143	H9A2···C5B^vii^	3.2826
C13A···H15A	3.2690	H9A2···C13A^xi^	3.4733
C13A···H17A	2.6943	H9A2···H1B^vii^	3.4365
C13A···H17B	2.8283	H9A2···H2B^vii^	3.3172
C13B···H9B1	2.6015	H9A2···H3B^vii^	3.3936
C13B···H9B2	2.9233	H9A2···H9A1^xi^	2.9945
C13B···H15B	3.2673	H9A2···H13A^xi^	2.5567
C13B···H17D	2.7230	H9A2···H17A^xi^	3.2760
C13B···H17E	2.7890	H9A2···H17B^xi^	3.4983
C14A···H16A	3.2596	H9B1···O1A	2.6898
C14A···H17A	2.6029	H9B1···C1A	2.9602
C14A···H17B	2.6673	H9B1···C5A	3.2785
C14A···H17C	3.2037	H9B1···C6A	3.2624
C14B···H16B	3.2545	H9B1···C9B^v^	3.5565
C14B···H17D	2.6222	H9B1···C13B^v^	3.2984
C14B···H17E	2.6492	H9B1···H1A^i^	3.5001
C14B···H17F	3.2050	H9B1···H6A	2.7650
C15A···H13A	3.2711	H9B1···H9B1^v^	3.1392
C15B···H13B	3.2702	H9B1···H9B2^v^	3.2489
C17A···H13A	2.5215	H9B1···H13B^v^	2.5684
C17B···H13B	2.5167	H9B1···H17D^v^	3.0913
H1A···H2A	2.2992	H9B2···N1A^v^	3.0336
H1B···H2B	2.2974	H9B2···C1A^v^	3.2985
H2A···H3A	2.3127	H9B2···C2A^v^	2.8904
H2B···H3B	2.3064	H9B2···C3A^v^	2.9447
H3A···H4A	2.3474	H9B2···C4A^v^	3.1532
H3B···H4B	2.3525	H9B2···C5A^v^	3.3625
H4A···H8A1	1.9890	H9B2···C13B^v^	3.5161
H4A···H8A2	2.6223	H9B2···H1A^v^	3.4543
H4B···H8B1	1.9888	H9B2···H2A^v^	3.3194
H4B···H8B2	2.6163	H9B2···H3A^v^	3.3943
H6A···H8A1	3.5813	H9B2···H9B1^v^	3.2489
H6B···H8B1	3.5854	H9B2···H13B^v^	2.5824
H8A1···H9A1	2.4195	H9B2···H17D^v^	3.0969
H8A1···H9A2	2.3025	H9B2···H17E^v^	3.3127
H8A2···H9A1	2.3013	H13A···O1B^iii^	3.5115
H8A2···H9A2	2.8577	H13A···N1B^x^	3.4617
H8B1···H9B1	2.4135	H13A···C9A^xi^	2.9967
H8B1···H9B2	2.3030	H13A···H1B^x^	3.2562
H8B2···H9B1	2.3020	H13A···H1B^iii^	3.3828
H8B2···H9B2	2.8566	H13A···H8A1^xi^	3.4374
H9A1···H13A	2.4265	H13A···H9A1^xi^	2.5694
H9A2···H13A	2.9957	H13A···H9A2^xi^	2.5567
H9B1···H13B	2.4275	H13B···O1A	3.5000
H9B2···H13B	3.0136	H13B···O1A^i^	3.5656
H13A···H17A	2.2374	H13B···N1A	3.4371
H13A···H17B	2.3898	H13B···C1A	3.5271
H13A···H17C	3.4914	H13B···C9B^v^	3.0242
H13B···H17D	2.2690	H13B···H1A	3.2363
H13B···H17E	2.3465	H13B···H1A^i^	3.3392
H13B···H17F	3.4883	H13B···H9B1^v^	2.5684
H15A···H16A	2.3198	H13B···H9B2^v^	2.5824
H15B···H16B	2.3119	H15A···O1A^vii^	3.3651
O1A···H1A^i^	1.82 (3)	H15A···C17B^viii^	2.9481
O1A···H2A^i^	3.5344	H15A···H2A^viii^	2.9548
O1A···H8B2	2.9740	H15A···H8B2^vii^	2.9180
O1A···H9B1	2.6898	H15A···H17D^viii^	3.4425
O1A···H13B	3.5000	H15A···H17E^viii^	2.4894
O1A···H13B^i^	3.5656	H15A···H17F^viii^	2.5707
O1A···H15A^vii^	3.3651	H15B···O1B^vi^	3.1705
O1A···H17E^i^	2.4719	H15B···C17A^xii^	2.9788
O1B···H1B^ii^	1.80 (3)	H15B···H1B^ix^	3.5086
O1B···H2B^ii^	3.5356	H15B···H2B^ix^	2.9363
O1B···H8A2^iv^	2.9375	H15B···H6B^vi^	3.5873
O1B···H9A1^iv^	2.7271	H15B···H8A2^v^	2.7849
O1B···H13A^iii^	3.5115	H15B···H17A^xii^	3.5094
O1B···H15B^vi^	3.1705	H15B···H17B^xii^	2.3967
O1B···H17B^iii^	2.4912	H15B···H17C^xii^	2.7158
O2A···H3B	2.9423	H16A···O2A^vii^	3.4235
O2A···H3B^vii^	3.3296	H16A···C3B	3.1966
O2A···H4B	2.5865	H16A···H3B	2.3163
O2A···H8B1	3.0557	H16A···H17F^viii^	2.7900
O2A···H16A^vii^	3.4235	H16B···O2B^vi^	3.1853
O2A···H17D^v^	2.6679	H16B···C3A^iv^	3.2609
O2A···H17F^v^	3.1116	H16B···C17A^xii^	3.5415
O2B···H3A^iv^	2.8622	H16B···H3A^iv^	2.3352
O2B···H3A^v^	3.4629	H16B···H6B^vi^	3.5601
O2B···H4A^iv^	2.5858	H16B···H17B^xii^	3.4993
O2B···H8A1^iv^	3.1964	H16B···H17C^xii^	2.7607
O2B···H16B^vi^	3.1853	H17A···O2B^vii^	2.6864
O2B···H17A^vii^	2.6864	H17A···C6B^vii^	3.2234
O2B···H17C^vii^	3.1185	H17A···C7B^vii^	3.2767
O3A···H2A^viii^	2.3436	H17A···C8A^xi^	3.3019
O3A···H8B2^vii^	3.2863	H17A···C9A^xi^	3.4878
O3B···H2B^ix^	2.3441	H17A···C12B^vii^	3.0581
O3B···H8A2^v^	3.2070	H17A···H4A^xi^	3.3142
N1A···H1A^i^	2.93 (3)	H17A···H6B^vii^	2.9790
N1A···H9B2^v^	3.0336	H17A···H8A1^xi^	2.4631
N1A···H13B	3.4371	H17A···H9A1^xi^	3.2732
N1A···H17E	3.3986	H17A···H9A2^xi^	3.2760
N1B···H1B^ii^	2.92 (3)	H17A···H15B^xiii^	3.5094
N1B···H9A2^vii^	2.9853	H17B···O1B^iii^	2.4912
N1B···H13A^iv^	3.4617	H17B···N1B^x^	3.5354
N1B···H17B^iv^	3.5354	H17B···C1B^iii^	3.2559
C1A···H1A^i^	2.71 (3)	H17B···C15B^xiii^	3.2749
C1A···H9B1	2.9602	H17B···H1B^x^	2.9558
C1A···H9B2^v^	3.2985	H17B···H8A1^xi^	3.3408
C1A···H13B	3.5271	H17B···H9A2^xi^	3.4983
C1A···H17E^i^	3.2226	H17B···H15B^xiii^	2.3967
C1B···H1B^ii^	2.70 (3)	H17B···H16B^xiii^	3.4993
C1B···H9A1^iv^	2.9439	H17C···O2B^vii^	3.1185
C1B···H9A2^vii^	3.2133	H17C···C2A^viii^	3.5271
C1B···H17B^iii^	3.2559	H17C···C3A^viii^	3.5886
C2A···H9B2^v^	2.8904	H17C···C12B^vii^	3.4482
C2A···H17C^ix^	3.5271	H17C···C15B^xiii^	3.2706
C2B···H9A2^vii^	2.8657	H17C···C16B^xiii^	3.2954
C2B···H17F^viii^	3.5513	H17C···H2A^viii^	2.9064
C3A···H9B2^v^	2.9447	H17C···H3A^viii^	3.0415
C3A···H16B^x^	3.2609	H17C···H8A1^xi^	3.5252
C3A···H17C^ix^	3.5886	H17C···H15B^xiii^	2.7158
C3B···H9A2^vii^	2.9221	H17C···H16B^xiii^	2.7607
C3B···H16A	3.1966	H17D···O2A^v^	2.6679
C4A···H9B2^v^	3.1532	H17D···C6A^v^	3.1614
C4B···H9A2^vii^	3.1002	H17D···C7A^v^	3.2187
C5A···H9B1	3.2785	H17D···C8B^v^	3.2661
C5A···H9B2^v^	3.3625	H17D···C9B^v^	3.3347
C5B···H9A1^iv^	3.2986	H17D···C12A^v^	3.0137
C5B···H9A2^vii^	3.2826	H17D···H4B^v^	3.4945
C6A···H9B1	3.2624	H17D···H6A^v^	2.9212
C6A···H17D^v^	3.1614	H17D···H8B1^v^	2.4760
C6B···H9A1^iv^	3.3610	H17D···H9B1^v^	3.0913
C6B···H17A^vii^	3.2234	H17D···H9B2^v^	3.0969
C7A···H3B^vii^	3.4330	H17D···H15A^ix^	3.4425
C7A···H17D^v^	3.2187	H17E···O1A^i^	2.4719
C7B···H3A^v^	3.5482	H17E···N1A	3.3986
C7B···H17A^vii^	3.2767	H17E···C1A^i^	3.2226
C8A···H6B^x^	3.2921	H17E···C15A^ix^	3.3979
C8A···H17A^xi^	3.3019	H17E···H1A	2.8349
C8B···H6A	3.2615	H17E···H2A	3.5542
C8B···H17D^v^	3.2661	H17E···H8B1^v^	3.3942
C9A···H6B^x^	3.5559	H17E···H9B2^v^	3.3127
C9A···H9A1^xi^	3.3310	H17E···H15A^ix^	2.4894
C9A···H13A^xi^	2.9967	H17F···O2A^v^	3.1116
C9A···H17A^xi^	3.4878	H17F···C2B^ix^	3.5513
C9B···H6A	3.4562	H17F···C12A^v^	3.4151
C9B···H9B1^v^	3.5565	H17F···C15A^ix^	3.2020
C9B···H13B^v^	3.0242	H17F···C16A^ix^	3.3113
C9B···H17D^v^	3.3347	H17F···H2B^ix^	2.9233
C10A···H1B^iii^	3.36 (3)	H17F···H3B^ix^	3.1267
C10A···H4B^vii^	3.3067	H17F···H8B1^v^	3.4716
C10B···H1A^i^	3.36 (3)	H17F···H15A^ix^	2.5707
C10B···H4A^v^	3.3216	H17F···H16A^ix^	2.7900
			
C14A—O3A—C17A	117.87 (17)	C1A—N1A—H1A	116.4 (16)
C14B—O3B—C17B	118.04 (17)	C2A—N1A—H1A	118.6 (16)
C1A—N1A—C2A	124.77 (18)	C1B—N1B—H1B	117.6 (16)
C1B—N1B—C2B	124.09 (19)	C2B—N1B—H1B	118.2 (16)
O1A—C1A—N1A	119.19 (18)	N1A—C2A—H2A	120.047
O1A—C1A—C5A	124.94 (19)	C3A—C2A—H2A	120.062
N1A—C1A—C5A	115.9 (2)	N1B—C2B—H2B	119.577
O1B—C1B—N1B	119.22 (18)	C3B—C2B—H2B	119.570
O1B—C1B—C5B	124.91 (19)	C2A—C3A—H3A	120.562
N1B—C1B—C5B	115.87 (19)	C4A—C3A—H3A	120.571
N1A—C2A—C3A	119.9 (2)	C2B—C3B—H3B	120.798
N1B—C2B—C3B	120.9 (2)	C4B—C3B—H3B	120.786
C2A—C3A—C4A	118.9 (3)	C3A—C4A—H4A	118.985
C2B—C3B—C4B	118.4 (3)	C5A—C4A—H4A	118.981
C3A—C4A—C5A	122.0 (2)	C3B—C4B—H4B	118.977
C3B—C4B—C5B	122.0 (2)	C5B—C4B—H4B	118.991
C1A—C5A—C4A	118.51 (19)	C5A—C6A—H6A	114.725
C1A—C5A—C6A	115.3 (2)	C7A—C6A—H6A	114.724
C4A—C5A—C6A	126.10 (19)	C5B—C6B—H6B	114.869
C1B—C5B—C4B	118.65 (19)	C7B—C6B—H6B	114.869
C1B—C5B—C6B	115.41 (19)	C7A—C8A—H8A1	108.953
C4B—C5B—C6B	125.82 (19)	C7A—C8A—H8A2	108.949
C5A—C6A—C7A	130.6 (2)	C9A—C8A—H8A1	108.951
C5B—C6B—C7B	130.3 (2)	C9A—C8A—H8A2	108.949
C6A—C7A—C8A	126.42 (19)	H8A1—C8A—H8A2	107.768
C6A—C7A—C12A	116.3 (2)	C7B—C8B—H8B1	108.959
C8A—C7A—C12A	117.25 (17)	C7B—C8B—H8B2	108.961
C6B—C7B—C8B	126.74 (19)	C9B—C8B—H8B1	108.958
C6B—C7B—C12B	116.4 (2)	C9B—C8B—H8B2	108.957
C8B—C7B—C12B	116.84 (18)	H8B1—C8B—H8B2	107.760
C7A—C8A—C9A	113.13 (19)	C8A—C9A—H9A1	109.091
C7B—C8B—C9B	113.11 (19)	C8A—C9A—H9A2	109.092
C8A—C9A—C10A	112.54 (19)	C10A—C9A—H9A1	109.085
C8B—C9B—C10B	112.72 (19)	C10A—C9A—H9A2	109.087
C9A—C10A—C11A	120.40 (18)	H9A1—C9A—H9A2	107.832
C9A—C10A—C13A	119.5 (2)	C8B—C9B—H9B1	109.041
C11A—C10A—C13A	119.94 (19)	C8B—C9B—H9B2	109.049
C9B—C10B—C11B	120.00 (18)	C10B—C9B—H9B1	109.055
C9B—C10B—C13B	119.72 (19)	C10B—C9B—H9B2	109.048
C11B—C10B—C13B	120.15 (18)	H9B1—C9B—H9B2	107.811
C10A—C11A—C12A	121.74 (18)	C10A—C13A—H13A	120.007
C10A—C11A—C16A	119.34 (19)	C14A—C13A—H13A	120.000
C12A—C11A—C16A	118.8 (2)	C10B—C13B—H13B	120.131
C10B—C11B—C12B	121.83 (18)	C14B—C13B—H13B	120.134
C10B—C11B—C16B	118.97 (19)	C14A—C15A—H15A	120.013
C12B—C11B—C16B	119.2 (2)	C16A—C15A—H15A	120.010
O2A—C12A—C7A	121.79 (19)	C14B—C15B—H15B	119.841
O2A—C12A—C11A	120.46 (19)	C16B—C15B—H15B	119.843
C7A—C12A—C11A	117.75 (19)	C11A—C16A—H16A	119.693
O2B—C12B—C7B	121.47 (19)	C15A—C16A—H16A	119.690
O2B—C12B—C11B	120.49 (18)	C11B—C16B—H16B	119.642
C7B—C12B—C11B	118.04 (19)	C15B—C16B—H16B	119.641
C10A—C13A—C14A	120.0 (2)	O3A—C17A—H17A	109.475
C10B—C13B—C14B	119.7 (2)	O3A—C17A—H17B	109.477
O3A—C14A—C13A	124.7 (2)	O3A—C17A—H17C	109.471
O3A—C14A—C15A	115.17 (19)	H17A—C17A—H17B	109.471
C13A—C14A—C15A	120.1 (2)	H17A—C17A—H17C	109.464
O3B—C14B—C13B	124.3 (2)	H17B—C17A—H17C	109.469
O3B—C14B—C15B	115.61 (19)	O3B—C17B—H17D	109.470
C13B—C14B—C15B	120.1 (2)	O3B—C17B—H17E	109.468
C14A—C15A—C16A	120.0 (3)	O3B—C17B—H17F	109.473
C14B—C15B—C16B	120.3 (2)	H17D—C17B—H17E	109.466
C11A—C16A—C15A	120.6 (3)	H17D—C17B—H17F	109.473
C11B—C16B—C15B	120.7 (3)	H17E—C17B—H17F	109.478
			
C17A—O3A—C14A—C13A	−0.9 (4)	C8B—C7B—C12B—O2B	−169.3 (2)
C17A—O3A—C14A—C15A	179.70 (19)	C8B—C7B—C12B—C11B	10.0 (3)
C17B—O3B—C14B—C13B	−1.2 (4)	C12B—C7B—C8B—C9B	−38.7 (3)
C17B—O3B—C14B—C15B	179.56 (19)	C7A—C8A—C9A—C10A	48.0 (3)
C1A—N1A—C2A—C3A	1.6 (4)	C7B—C8B—C9B—C10B	48.6 (3)
C2A—N1A—C1A—O1A	−178.9 (2)	C8A—C9A—C10A—C11A	−30.5 (3)
C2A—N1A—C1A—C5A	−0.2 (4)	C8A—C9A—C10A—C13A	153.73 (18)
C1B—N1B—C2B—C3B	1.2 (4)	C8B—C9B—C10B—C11B	−31.3 (3)
C2B—N1B—C1B—O1B	−178.9 (2)	C8B—C9B—C10B—C13B	152.79 (18)
C2B—N1B—C1B—C5B	0.6 (4)	C9A—C10A—C11A—C12A	0.7 (4)
O1A—C1A—C5A—C4A	176.6 (2)	C9A—C10A—C11A—C16A	−176.02 (19)
O1A—C1A—C5A—C6A	−0.5 (4)	C9A—C10A—C13A—C14A	175.01 (18)
N1A—C1A—C5A—C4A	−2.1 (3)	C11A—C10A—C13A—C14A	−0.8 (4)
N1A—C1A—C5A—C6A	−179.12 (17)	C13A—C10A—C11A—C12A	176.46 (19)
O1B—C1B—C5B—C4B	176.5 (2)	C13A—C10A—C11A—C16A	−0.3 (4)
O1B—C1B—C5B—C6B	0.4 (4)	C9B—C10B—C11B—C12B	2.2 (4)
N1B—C1B—C5B—C4B	−2.9 (3)	C9B—C10B—C11B—C16B	−175.18 (18)
N1B—C1B—C5B—C6B	−179.10 (18)	C9B—C10B—C13B—C14B	174.82 (18)
N1A—C2A—C3A—C4A	−0.5 (4)	C11B—C10B—C13B—C14B	−1.0 (4)
N1B—C2B—C3B—C4B	−0.7 (4)	C13B—C10B—C11B—C12B	178.08 (19)
C2A—C3A—C4A—C5A	−1.9 (4)	C13B—C10B—C11B—C16B	0.7 (4)
C2B—C3B—C4B—C5B	−1.8 (4)	C10A—C11A—C12A—O2A	−169.6 (2)
C3A—C4A—C5A—C1A	3.2 (4)	C10A—C11A—C12A—C7A	11.0 (4)
C3A—C4A—C5A—C6A	179.8 (2)	C10A—C11A—C16A—C15A	0.7 (4)
C3B—C4B—C5B—C1B	3.6 (4)	C12A—C11A—C16A—C15A	−176.1 (2)
C3B—C4B—C5B—C6B	179.3 (2)	C16A—C11A—C12A—O2A	7.2 (4)
C1A—C5A—C6A—C7A	−163.1 (2)	C16A—C11A—C12A—C7A	−172.2 (2)
C4A—C5A—C6A—C7A	20.2 (4)	C10B—C11B—C12B—O2B	−171.6 (2)
C1B—C5B—C6B—C7B	−162.5 (2)	C10B—C11B—C12B—C7B	9.2 (3)
C4B—C5B—C6B—C7B	21.6 (4)	C10B—C11B—C16B—C15B	0.0 (4)
C5A—C6A—C7A—C8A	1.5 (4)	C12B—C11B—C16B—C15B	−177.4 (2)
C5A—C6A—C7A—C12A	−177.9 (2)	C16B—C11B—C12B—O2B	5.8 (4)
C5B—C6B—C7B—C8B	0.6 (4)	C16B—C11B—C12B—C7B	−173.4 (2)
C5B—C6B—C7B—C12B	−178.0 (2)	C10A—C13A—C14A—O3A	−178.1 (2)
C6A—C7A—C8A—C9A	142.6 (3)	C10A—C13A—C14A—C15A	1.3 (4)
C6A—C7A—C12A—O2A	8.7 (4)	C10B—C13B—C14B—O3B	−178.5 (2)
C6A—C7A—C12A—C11A	−171.92 (19)	C10B—C13B—C14B—C15B	0.7 (4)
C8A—C7A—C12A—O2A	−170.8 (2)	O3A—C14A—C15A—C16A	178.6 (2)
C8A—C7A—C12A—C11A	8.6 (3)	C13A—C14A—C15A—C16A	−0.9 (4)
C12A—C7A—C8A—C9A	−37.9 (3)	O3B—C14B—C15B—C16B	179.3 (2)
C6B—C7B—C8B—C9B	142.7 (3)	C13B—C14B—C15B—C16B	0.0 (4)
C6B—C7B—C12B—O2B	9.5 (4)	C14A—C15A—C16A—C11A	−0.2 (4)
C6B—C7B—C12B—C11B	−171.26 (19)	C14B—C15B—C16B—C11B	−0.4 (4)

Symmetry codes: (i) −*x*+3, −*y*+1, −*z*; (ii) −*x*+1, −*y*+2, −*z*+1; (iii) −*x*+1, −*y*+1, −*z*+1; (iv) *x*, *y*+1, *z*; (v) −*x*+2, −*y*+1, −*z*; (vi) −*x*+2, −*y*+2, −*z*; (vii) −*x*+2, −*y*+1, −*z*+1; (viii) *x*−1, *y*, *z*+1; (ix) *x*+1, *y*, *z*−1; (x) *x*, *y*−1, *z*; (xi) −*x*+2, −*y*, −*z*+1; (xii) *x*+1, *y*+1, *z*−1; (xiii) *x*−1, *y*−1, *z*+1.

### (II) 3-[(E)-(6-Methoxy-1-oxo-1,2,3,4-tetrahydronaphthalen-2-ylidene)methyl]pyridin-2(1H)-one Hydrogen-bond geometry (Å, º)

**Table:**

*D*—H···*A*	*D*—H	H···*A*	*D*···*A*	*D*—H···*A*
N1*A*—H1*A*···O1*A*^i^	0.96 (3)	1.82 (3)	2.778 (3)	178 (3)
N1*B*—H1*B*···O1*B*^ii^	0.98 (3)	1.80 (3)	2.778 (3)	176 (3)

Symmetry codes: (i) −*x*+3, −*y*+1, −*z*; (ii) −*x*+1, −*y*+2, −*z*+1.
